# Microstructure Evolution and Mechanical Properties of 7A52 Aluminum Alloy Thin Sheet Repaired with Friction Stir Surfacing

**DOI:** 10.3390/ma17112602

**Published:** 2024-05-28

**Authors:** Xiangxue Li, Chengcheng Shi, Guofeng Han, Huan Liu, Xiaofei Li, Rui Liu

**Affiliations:** 1 School of Mechanical Engineering, Shandong University of Technology, Zibo 255000, China; lixiangxue19990118@163.com (X.L.); liuhuan000824@163.com (H.L.); lixiaofei20210629@163.com (X.L.); 19862512385@163.com (R.L.); 2 National Key Lab for Remanufacturing, Beijing 100072, China; faf428@sina.com

**Keywords:** surface friction welding, 7A52 aluminum alloy, damage repair, microstructure, mechanical properties

## Abstract

A solid-state repair technique based on surface friction welding is investigated in depth to achieve excellent mechanical properties of damaged 7A52 aluminum alloy. The results show that the yield strength and tensile strength along the repair direction are 436 MPa and 502 MPa, respectively, at a rotational speed of 1400 rpm and a travel speed of 300 mm/min, which are about 157.9% and 129.7% of those before the defects were repaired, respectively, while the elongation is 17.2% compared to the base material. Perpendicular to the repair direction, the yield strength and tensile strength are 254 MPa and 432 MPa, which are 111.4% and 129.7% of those before the defects were repaired, respectively, while the elongation is 11.8% compared to the base material. The mechanical properties of the repaired areas are still improved compared to those of the defect-free sheets. On the one hand, this is attributed to the dynamic recrystallization of the nugget zone due to the thermo-mechanical coupling, resulting in the formation of a fine, equiaxed grain structure; on the other hand, the precipitated Mg_2_Si phase, which is incoherent within the base material, transforms into the Al_12_(Fe, Mn)_3_Si phase, as well as the precipitation of the Al_6_Mn phase and η′ phase, resulting in the enhancement of the properties. The material fracture at the junction of the nugget zone and the heat-affected zone occurs after repair, which is attributed to the significant difference in the texture of the nugget zone and the heat-affected zone, as well as to the stress concentration at the junction.

## 1. Introduction

Because of their excellent comprehensive mechanical properties, 7xxx series aluminum alloys are widely used in the field of special vehicles and ships [1,2,3,4,5,6]. However, these alloys are prone to failures such as corrosion and shallow cracking during long-term employment; when repaired with traditional fusion welding methods, defects such as thermal cracks, pores, and inclusions are generated universally [7,8,9,10], leading to poor post-repair performance, which cannot meet the demands of applications. Therefore, research has been devoted to the realization of these alloys’ excellent repair properties.

The friction stir welding (FSW) principle can achieve superior weld morphology and mechanical properties and is widely used in aluminum alloy welding due to its high efficiency, simple operation, and absence of porosity and cracks [2,3,7,8,9,10,11,12,13,14]. For example, Huang [1] and Feng et al. [15] obtained excellent mechanical properties of the joints with the friction stir welding of 7A52 aluminum alloys, although there was a pin-hole defect at the end of the welding channel.

However, conventional friction stir repair cannot avoid pin-hole defects and does not provide sufficient integrity and performance for shallow cracks. As a new method of friction stir welding, surface friction welding [16], also known as pinless friction welding, can achieve pin-hole free welding of sheets and has become a hotspot in the field of aluminum alloy welding in recent years. Y. Ni [17] investigated the surface friction repair technique as applied to an AA7075 aluminum alloy, and the results showed that the tensile strength could be 482 MPa, which was 90% relative to the base material. Yazdi et al. [14] showed that after repairing aluminum alloys with different tip shapes of stirring heads, the mechanical properties of the joints obtained with the “pinless” tool are higher than those of the “pinned” tool, due to the higher contact surface area of the “pinless” stirring head.

However, current research mainly focuses on the welding properties of aluminum alloys, and there is still a lack of research on the surface friction repair of 7xxx series aluminum alloy sheets. Given this, this study adopts surface friction welding repair technology to repair cracks on 7A52 aluminum alloys and explores in detail the influence of key process parameters such as stirring speed and travel speed on the repair integrity of surface crack defects, microstructure, and mechanical properties of 7A52. The aims of this study are to reveal the mechanism of toughening and establish a basis for the application of pinless friction welding repair in 7A52 aluminum alloys.

## 2. Materials and Methods

### 2.1. Materials

**Figure 1:**
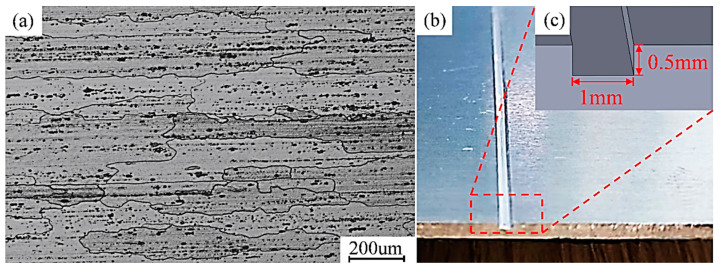
(**a**) Microstructure of the base material; (**b**) macrograph of the defects; (**c**) size of the defects.

The experimental material was a 2 mm 7A52 aluminum alloy sheet; the composition of the material is shown in Table 1 and its microstructure is shown in Figure 1a. The heat treatment state was T6. In order to simulate the crack damage on the surface of the material, a groove with a cross-section of 0.5 mm (depth) and 1 mm (width) was milled. The micromorphology and dimensions are shown in Figure 1b,c.

### 2.2. Methods

**Figure 2:**
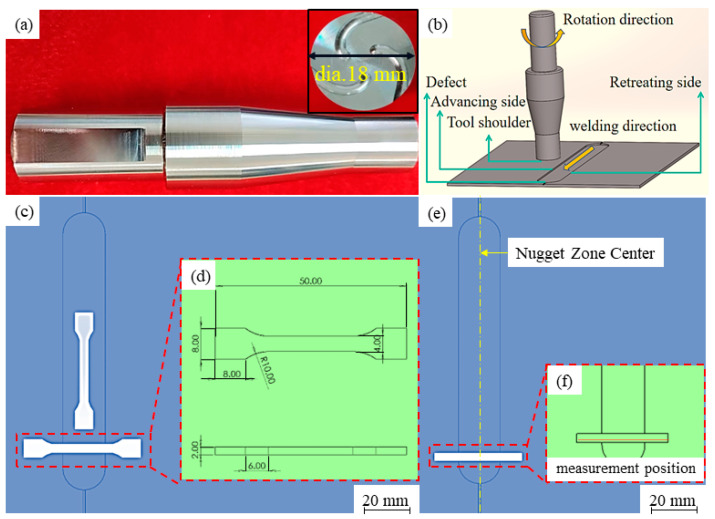
(**a**) Morphology of the stirring head; (**b**) repair process; (**c**,**d**) sample location and size of tensile test specimens; (**e**,**f**) sample location of hardness measurement.

The surface friction repair process parameters are shown in Table 2. The morphology of the stirring head is shown in Figure 2a, the material of the repair tool is H13 tool steel, the diameter of the end face is 18 mm, and there are three involute spherical grooves uniformly distributed on the end face. The repair process is shown in Figure 2b. The thickness was measured several times in the repaired area with different parameters and the average value was taken as the calculated value of the reduction rate. The repair experimental equipment model is TR-FSW-T300 (Weihai, China). After that, a rectangular sample was cut along the direction perpendicular to the repair for microstructure observation, ground with sandpaper, and placed on a metallographic polishing machine. Keller’s reagent (2.5 mL HNO_3_ + 1.5 mL HCl + 1 mL HF + 95 mL H_2_O) was used to etch the surface of the microstructure to observe the grain boundaries (Zibo, Shandong Province, China). Vickers hardness was measured by using an HXD-2000TM/LCD (Shanghai, China) microhardness tester with a downward pressure load of 50 gf and a dwell time of 15 s. The morphology of the fracture was observed using Scanning Electron Microscopy (SEM). The Electron Backscatter Diffraction (EBSD) observation samples were taken from the advancing side of the repair area, after mechanical polishing of the specimens followed by electropolishing. The composition of the electrolyte was perchloric acid/alcohol at a ratio of 1:3, the power supply voltage was 20 V, and the electrolytic polishing time was 8 s. The Transmission Electron Microscopy (TEM) samples were taken from the center of the repaired area after mechanical polishing and ion milling, and then observed using an FEI-Talos F200S Transmission Electron Microscope (Waltham, MA, USA), with the accelerating voltage selected as 200 kV. The sample location and size of the tensile test specimens and hardness measurement are shown in Figure 2c–f.

## 3. Results and Discussion

### 3.1. Effect of Process Parameters on Reduction Rate

**Figure 3:**
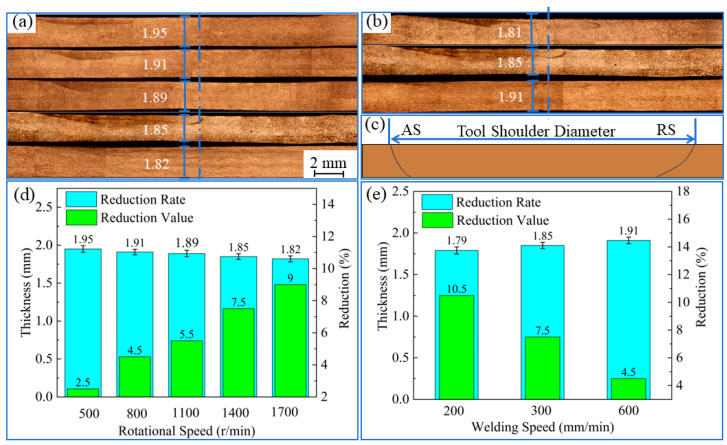
Macrograph of the thickness at the center of the nugget zone and its reduction rate histograms for different parameters: (**a**) rotational speed is 500 rpm, 800 rpm, 1100 rpm, 1400 rpm, and 1700 rpm; (**d**) its thickness and the histogram of the reduction rate; (**b**) travel speed is 200 mm/min, 300 mm/min, or 600 mm/min; (**e**) its thickness and the histogram of the reduction rate; (**c**) location schematic of the repaired area.

The repaired area’s thickness should not normally be reduced by more than 10% of the base material; otherwise, the performance suffers significantly. Yan provided an equation for evaluating the reduction in the repair area, known as the reduction rate [18]. Figure 3 shows a macrograph of the thickness at the center of the nugget zone and its thinning rate histogram for different parameters. When the travel speed is constant, with an increase in the rotational speed, the friction heat between the shoulder and the material increases, the plastic flow of the material is better, and the loss of material is more serious even under the same top pressure, which results in a gradual increase in the reduction rate. When the rotational speed is constant, with an increase in the travel speed, the residence time of the shoulder at each place of the weld channel is decreased. The material is affected by the frictional heat for a shorter period of time; therefore, the plastic deformation ability of the material is gradually reduced, and the loss of material is reduced, so the rate of reduction decreases gradually [19].

### 3.2. Microstructure Evolution

**Figure 4:**
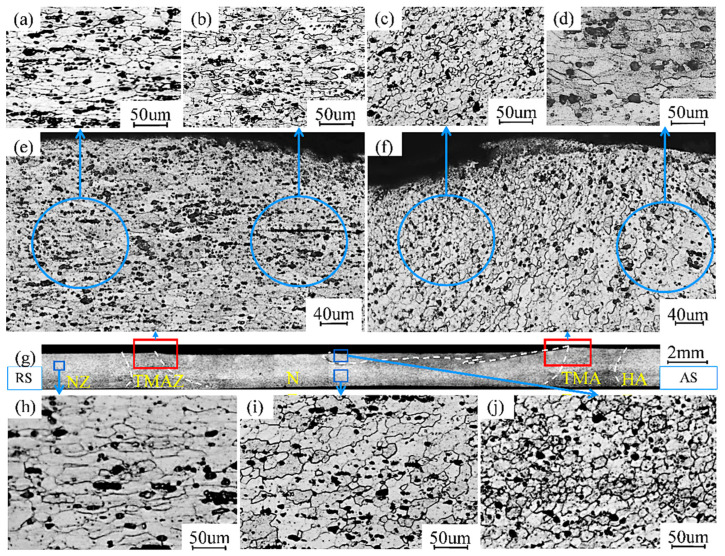
Microstructure of different positions of the cross-section based on the parameters 1400 rpm and 300 mm/min.

The cross-sectional area of the repair area observed using a metallographic microscope with repair parameters of 1400 rpm and 300 mm/min and the microstructure of its advancing and retreating sides are shown in Figure 4. Similar to conventional FSW, the repair area can be divided into four zones [8]: base material (BM), nugget zone (NZ), thermo-mechanically affected zone (TMAZ), and heat-affected zone (HAZ).

The grain structure of the base material shows a fibrous shape and is arranged along the rolling direction. The material in the NZ undergoes intense plastic deformation due to the direct stirring action of the stirring head, which experiences high thermal cycling. The grains grow upwards with continuous disruption and undergo dynamic recrystallization, leading to the formation of a fine, equiaxed and recrystallized structure [20,21]. Figure 4g shows a cross-section in the repair direction, with the advancing side on the right. Figure 4e,f show the magnification of the junction of the NZ and HAZ on the receding side (RS) and advancing side (AS), respectively. It can easily be seen that the boundary junction of the NZ and HAZ on the AS is more clearly defined compared to the RS, and the HAZ region is wider on the AS compared to the RS [22].

Comparing Figure 4a–d, the grain structure is found to be finer on the NZ side as compared to the HAZ side. Stratification can be observed in the NZ region close to the AS, and the upper NZ and the lower NZ are shown in Figure 4i,j, respectively. The material also exists at a 10% flow rate in the sheet thickness direction due to the inclination angle and top pressure, where the upper material moves towards the advancing side and the lower material moves towards the receding side. This difference in grain structure between the upper and lower parts of the NZ region can be explained by the difference in plastic material flow behaviors and thermal effects in the different regions: The material on the retreating side moves backwards and only a small part moves forward. The high linear velocity on the forward side generates more frictional heat, leading to differences in their properties. Moreover, there is a sheet surface oxide layer that enters the material inside with the stirring action, which makes the delamination phenomenon more obvious [23].

**Figure 5:**
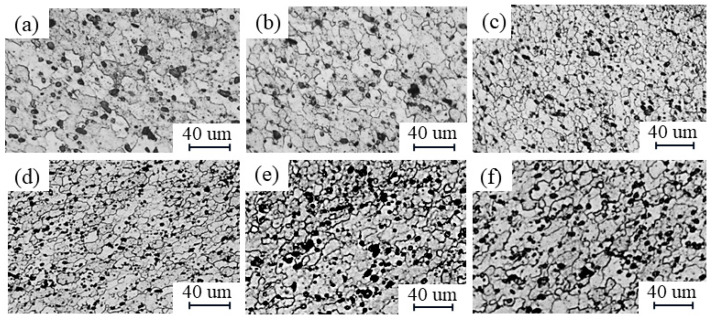
Comparison of NZ grain size according to different parameters: (**a**–**c**) travel speed is constant at 300 mm/min and rotational speeds are 500 rpm, 1100 rpm, and 1700 rpm, respectively; (**d**–**f**) rotational speed is constant at 800 rpm and travel speeds are 200 mm/min, 300 mm/min, and 600 mm/min, respectively.

**Figure 6:**
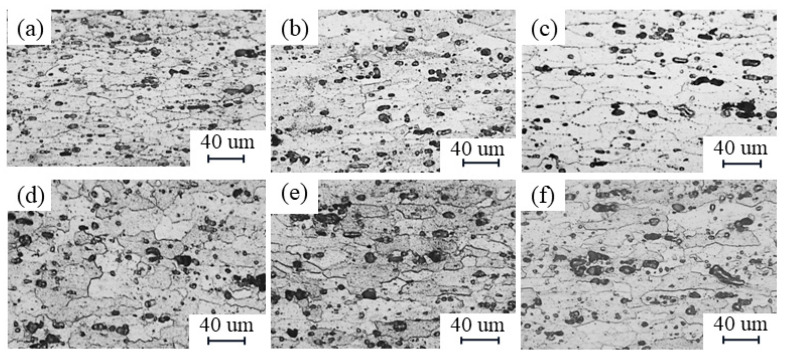
Comparison of HAZ grain size according to different parameters: (**a**–**c**) travel speed is constant at 300 mm/min and rotational speeds are 500 rpm, 1100 rpm, and 1700 rpm, respectively; (**d**–**f**) rotational speed is constant at 800 rpm and travel speeds are 200 mm/min, 300 mm/min, and 600 mm/min, respectively.

Figure 5 and Figure 6 show a comparison of the microstructure of the NZ and HAZ based on different parameters. When the travel speed is constant, with an increase in the rotational speed, the NZ grain size decreases and the degree of recrystallization increases. This is because a higher rotational speed intensifies the stirring action of the tool. The HAZ, on the other hand, increases the grain size and the grains are coarsened by heat, which is due to more frictional heat being generated from a higher rotational speed [24]. When the rotational speed is constant, the NZ grain size increases as the travel speed increases because the faster travel speed results in less tool residence time at any position, leading to less stirring, while the HAZ grain size decreases, which is due to less grain growth as the heat input is gradually reduced [4,25].

**Figure 7:**
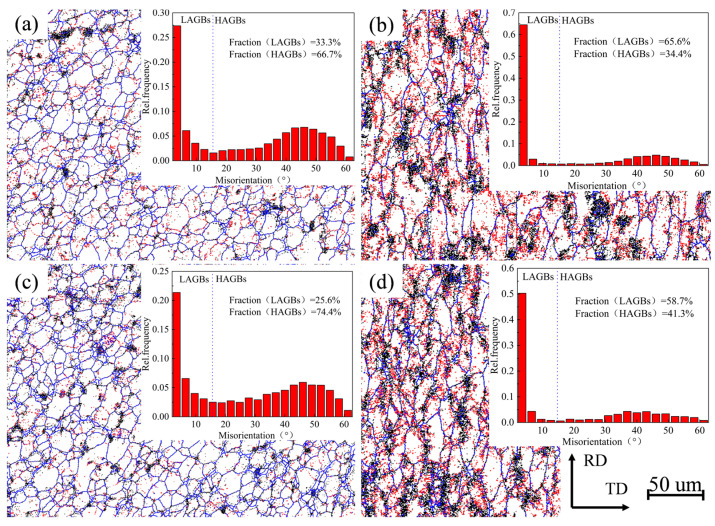
Differences in grain boundary images and histograms between NZ and HAZ with different parameters and histograms: (**a**,**b**) rotational speeds of 300 mm/min; (**c**,**d**) rotational speeds of 200 mm/min.

Figure 7 shows the grain boundary images for different parameters. When the moving speed is 300 mm/min, the proportion of HAGBs on the NZ is 66.7% and the proportion of LAGBs is 33.3%. When the moving speed is 200 mm/min, the proportion of HAGBs on the NZ is 74.4% and the proportion of LAGBs is 25.6%. The NZ is mainly composed of HAGBs, which show typical recrystallization characteristics. Moreover, as the travel speed decreases, the content of HAGBs increases and the content of LAGBs decreases, which means that there is a transformation of LAGBs to HAGBs after absorbing energy [26,27,28]. The proportion of HAGBs is 34.4% and the proportion of LAGBs is 65.6% when the moving speed is 300 mm/min, and the proportion of HAGBs is 41.3% and the proportion of LAGBs is 58.7% when the moving speed is 200 mm/min. In the NZ region, there is a significant transformation of its grain morphology from the fibrous shape to equiaxed grains, which is due to the occurrence of complete recrystallization. In contrast, the grain morphology in the HAZ is coarse and elongated with a large number of LAGBs, which is due to the growth of grains in the HAZ caused by the frictional heat instead of the stirring of the tool [29].

**Figure 8:**
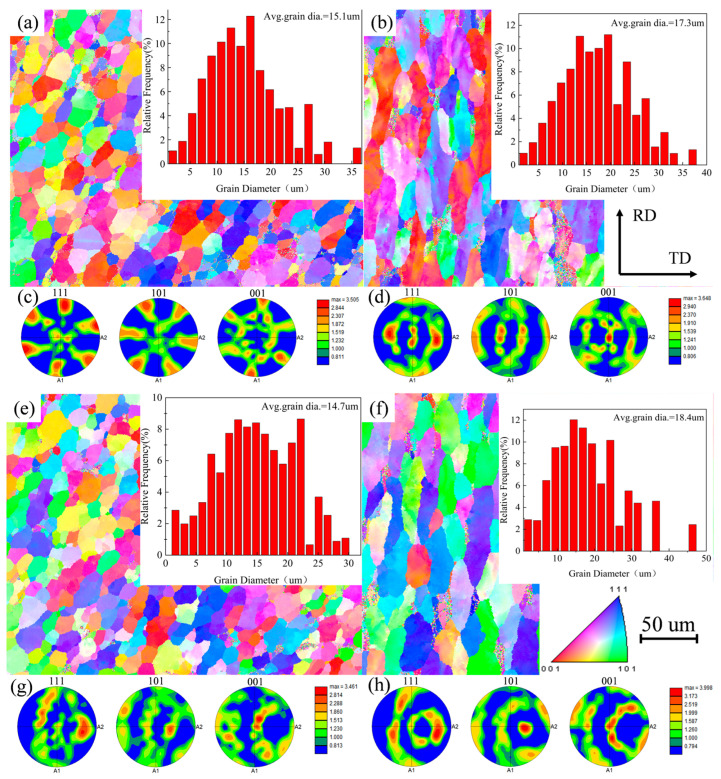
Microstructure, grain size distribution map, and polar plots of NZ and HAZ for different parameters: (**a**,**b**) travel speed 300 mm/min; (**c**,**d**) travel speed 200 mm/min.

Figure 8a,b show the IPF images and polar plots of the NZ and HAZ at a travel speed of 300 mm/min, with average grain sizes of 15.1 um and 17.3 um. Figure 8c,d show the IPF images and polar plots of the NZ and HAZ at travel speeds of 300 mm/min and 200 mm/min, with average grain sizes of 14.7 um and 18.4 um. Based on a comparison, it was found that the degree of refinement of the NZ grains is higher than that of 300 mm/min when the travel speed is 200 mm/min, while the HAZ grains are coarser, which is consistent with the above metallographic observations. From the figure, it can be seen that the lower travel speed improves the dynamic recrystallization and grain growth of the NZ, leading to a finer and more uniform microstructure of the grain structure in the repaired region [30]. Comparing the polar plots of different regions, it can be seen that the NZ grains do not have obvious orientation, which consists of equiaxed isotropic grains, and the strength of the texture decreases with the refinement of the grain structure and the increase in the degree of recrystallization. However, the grain structure of the HAZ grains has a similar orientation [30,31,32], the anisotropy of the grain texture is strong, and the strength of the texture gradually improves with a decrease in the travel speed. This can be explained by the degree of thermo-mechanical coupling in different regions.

**Figure 9:**
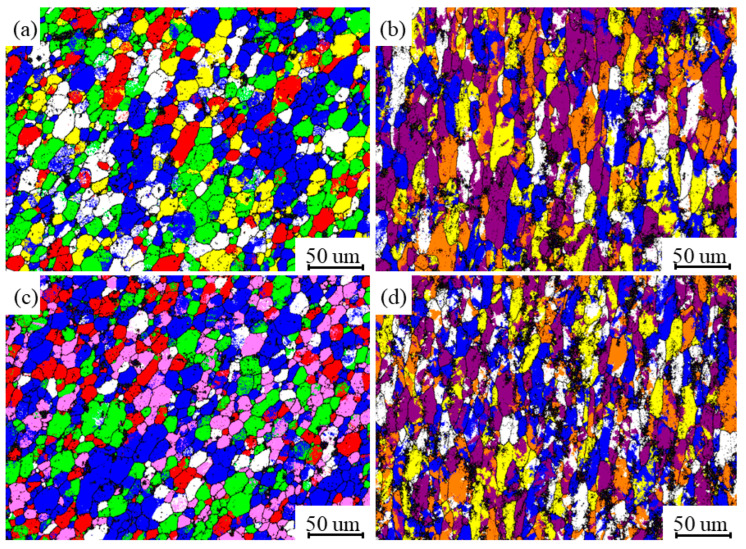
Texture distribution of NZ and HAZ weaves for different parameters: (**a**,**b**) travel speed is 300 mm/min; (**c**,**d**) travel speed is 200 mm/min.

Figure 9 shows the texture distribution of the NZ and HAZ for different parameters, and Table 3 shows the percentage of the main textures. Using Channel 5 software to analyze the texture, the grain color difference and distribution corresponding to different textures are shown in Figure 9 and Table 3. The relative percentages of the NZ recrystallization map texture {001} <110> and Goss texture at 300 mm/min are 29.4% and 23%, respectively, and the relative percentages of the HAZ residual rolling texture S {123} <634> and recrystallization texture {001} <110> are 31.6% and 19.8%, respectively. At 200 mm/min, the main texture types in the NZ are the recrystallization map texture {001} <110> and Copper texture, with relative contents of 37.5% and 20.6%, respectively, and the main texture types in the HAZ are the residual rolling texture S {123} <634> and recrystallization texture {001} <110>, with relative contents of 25.7% and 23.9%, respectively. The NZ forms a large number of recrystallized structures due to the thermo-mechanical coupling and the compression of the stirring head, while the HAZ not only contains some recrystallized structures but also some residual rolled textures due to the heat influence and partial stirring. Comparing the texture types and contents of different zones based on different parameters, it was found that the recrystallization texture {001} <110> in the NZ decreases with an increase in the travel speed, and the residual rolling texture S {123} <634> increases in the HAZ [27].

In summary, in the NZ, there is complete dynamic recrystallization, forming fine equiaxial grains; the HAZ forms coarse grains along the repair direction due to the thermo-mechanical coupling. According to the Hall–Petch formula, the smaller the grains, the higher the dislocation density between grain boundaries. Under the same stress, small grains require a larger applied stress in order to cause the plastic deformation of adjacent grains. In addition, the finer the grains, the more twisted the grain boundaries are, which is not conducive to crack propagation, and more energy can be absorbed in the fracture process. Therefore, with an increase in the degree of plastic deformation of the NZ, the dislocation density and dislocation motion resistance increase, resulting in a stress concentration phenomenon at its junction with the HAZ: the larger the difference in grain size, the greater the stress concentration, so the fracture location always occurs on the AS of the junction between the NZ and the HAZ. This is why the mechanical properties of the material perpendicular to the repair direction are reduced.

**Figure 10:**
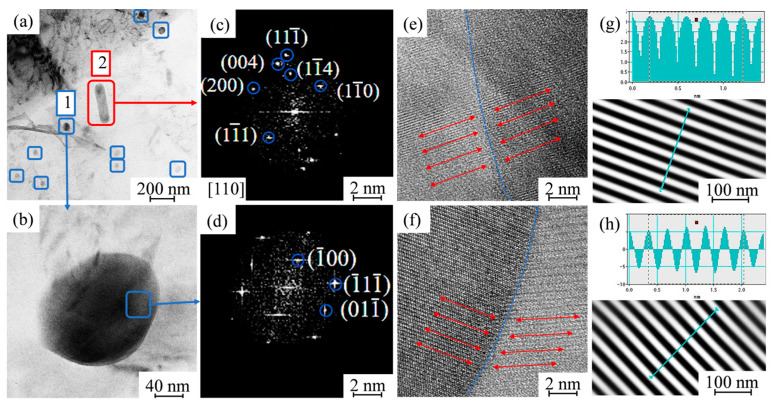
(**a**) Bright-field image of Al_6_Mn phase and Al_12_(Fe, Mn)_3_Si phase; (**b**) shows the morphology of the second phase 1. (**c**,**d**) diffraction spots of Al_6_Mn phase and Al_12_(Fe, Mn)_3_Si phase; (**e**,**f**) interface under high resolution; (**g**,**h**) measurement of their intergranular spacing.

Figure 10 shows a micrograph of the second phase, diffraction spots, high resolution at the grain boundaries, and crystal spacing. The energy spectrum analysis for the second phase 1 in Figure 10a reveals that it mainly consists of Al, Fe, Mn, and Si elements, and since these elements mainly exist as Al_12_(Fe, Mn)_3_Si compounds in the NZ, and according to the calibration of its diffraction spots, the circular precipitates in Figure 10b are found to be α-Al_12_(Fe, Mn)_3_Si. Observing the high resolution at the interface, measuring its crystal spacing and calculating the mismatch shows that the misfit is 0.05 < δ < 0.25, and that it is a semicoherent interface with the base material.

Figure 10c shows the electron diffraction pattern of the [110] zone axis of the second phase in combination with the α-Al [011] zone axis. According to the calibration of its diffraction spots, it is known that the second phase is Al_6_Mn; by observing the high resolution at the interface and calculating the mismatch degree, it is known that 0.05 < δ < 0.25, which is also a semicoherent interface. Related studies have shown that it can effectively obstruct the dislocation and grain boundary migration to improve the strength of the repaired area [1,3,33,34].

**Figure 11:**
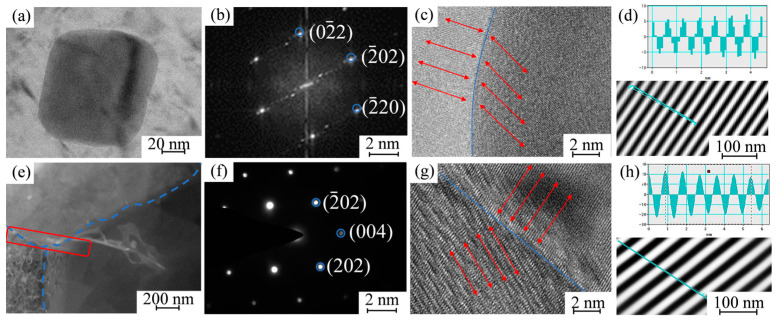
(**a**,**e**) Bright-field image of η′ phase and Mg_2_Si phase; (**b**,**f**) diffraction spots of MgZn_2_ phase and Mg_2_Si phase; (**c**,**g**) interface under high resolution; (**d**,**h**) measurement of interplanar spacing for the Mg_2_Si phase.

Figure 11 shows a micrograph of the MgZn_2_ phase and Mg_2_Si phase, the grain boundaries under high resolution and crystal spacing.

Relevant studies have shown that [35] the fine MgZn_2_ as the η′ phase is diffusely distributed inside the grains and grain boundaries, and by analyzing its grain spacing and mismatch, it can be seen that the semicoherent interface of the transition phase η′ is the main reinforcing phase, which is able to improve the hardness and strength of the alloy. It has been shown [34] that these two elements only exist as Mg_2_Si compounds in 7xxx series aluminum alloys, and the Mg_2_Si phase is diffusely distributed in the aluminum alloy base. However, there is not a significant amount of Mg_2_Si phase in the repaired area and only a small amount of it is found at the grain boundaries. Observing the high resolution at its interface and measuring its grain spacing shows that the mismatch is larger than 0.25, so the Mg_2_Si phase and the base phase are incoherent interfaces, which are prone to sprout cracks and reduce the performance of the material.

The incoherent Mg_2_Si phase is reduced by back dissolution, and its elements are selectively precipitated to form new chemosynthetic phases with other phases in the base. Combined with the above results and analyses of related studies, it can be seen that the strengthening mechanism of the NZ in the second phase is as follows: The Si element in the base material precipitates and the Al_6_(Fe, Mn) phase, which is coarse in the rolling direction, forms the α-Al_12_(Fe, Mn)_3_Si phase, while the Mg element is selectively dissolved. The NZ is strengthened due to the reduction of the deleterious phase as Mg_2_Si. The forming of Al_6_Mn is due to the fact that the Mn element has already formed a supersaturated solid solution in the base material, and when it is crystallized, it cannot precipitate out in time because of the fast cooling rate, and it exists in an unstable state in the α-Al. Accompanied by the temperature increase in the NZ during the stirred friction repair process, the Mn element precipitates out and exists in the alloy base material and grain boundaries in the form of Al_6_Mn compounds. The dual effect of grain refinement strengthening and precipitated phase refinement strengthening results in an increase in the strength and hardness of the NZ compared to the BM.

**Figure 12:**
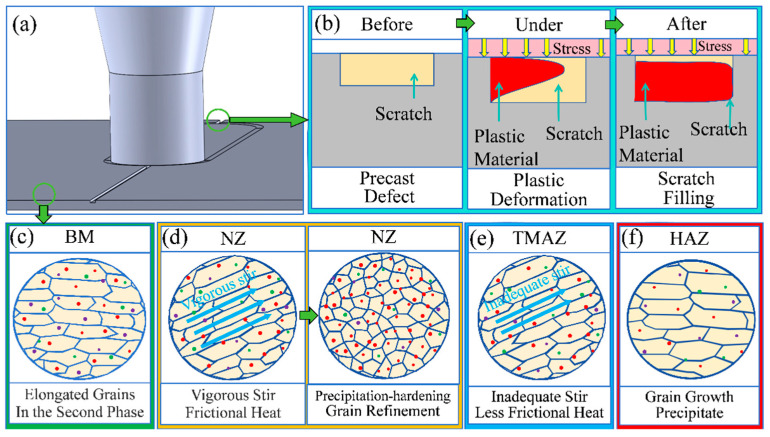
Schematic diagram of the repair process, defect-filling process, and microstructure evolution: (**a**) repair process; (**b**) material flow in filling; (**c**–**f**) microstructure evolution of BM, NZ, TMAZ, and HAZ, respectively.

Figure 12 shows a diagram of the filling process of the scratches and the structure evolution process in different regions during the repair process, and different-colored spots in the grain represent different second phases. Before the repair, the BM grains showed a fibrous shape along the rolling direction, and the middle of the sheet carried a defect with a rectangular groove. During the repair process, dramatic plastic deformation occurred in the NZ under the action of tool stirring, and the structure experienced sufficient dynamic recrystallization to form a fine equiaxial crystalline structure. Plastic flow occurred at the scratch due to the combined effect of thermal coupling, and the plastic material flowed along the direction of the tool linear velocity and gradually filled the defect. After repair, the HAZ is subjected to a small amount of mechanical stirring, and the grain structure is still in a state of elongated grains. When the HAZ is subjected to friction heat only, the grain structure grows abnormally as a result of the heat. Along with the top pressure applied by the tool, the plastic material fills the scratches under pressure and finally forms a complete dense structure [20].

Before repair, various second phases were diffusely distributed in the base. After the repair process, the NZ experienced strong thermo-mechanical effects, the composition of the precipitated phases changed dramatically, and the dislocation density increased. In addition, the recrystallization process led to an increase in the number of grain boundaries, which provided more locations for the precipitation of the second phases. In contrast, the HAZ is only affected by thermal cycling and undergoes a back dissolution of the second phase, resulting in a reduction in its content.

### 3.3. Mechanical Properties

**Figure 13:**
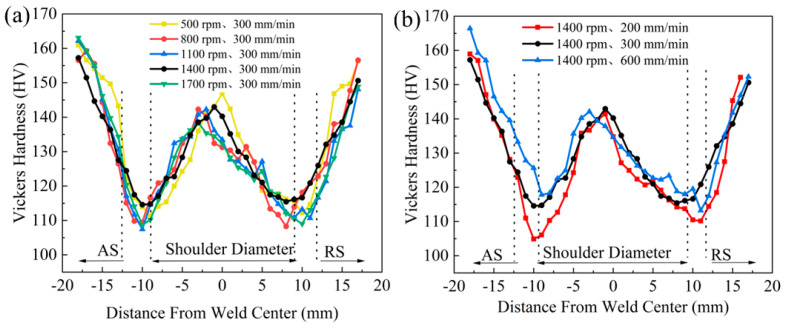
Vickers hardness distribution from the center to the sides of the repaired area for different parameters: (**a**) gradual increase in rotational speed; (**b**) gradual increase in travel speed.

Figure 13a,b show the Vickers hardness distributions along the center to both sides for different parameters. Similar to the traditional FSW hardness distribution law, the hardness distribution pattern shows a typical “W” shape: The hardness is higher on the two sides, decreases when approaching the repair area, and the minima are located at the junction of the NZ and HAZ on the two sides. The hardness gradually increases near the center, but it is always lower than the hardness of the base material [36].

The minimum value of the hardness in both experimental cases is located at the junction of the NZ and the HAZ on the advancing side, because the NZ experiences not only friction heat but also plastic deformation, which makes its hardness higher than that of the HAZ. By contrast, the HAZ only experiences the thermal cycle of friction heat but does not experience the violent stirring friction of the tool shoulder, which tends to cause coarseness of the grains, resulting in a lower hardness. Although the grain size of the NZ is smaller than that of the BM, the precipitation-hardening effect in this region is not as effective as that of the base material due to the second-phase dissolution, resulting in a lower hardness than that of the base material. In addition, from Figure 13a, it can be seen that with the increase in the rotational speed, the frictional heat received by the material increases and the hardness decreases as a whole. In Figure 13b, when the rotational speed of the tool is constant, the hardness gradually increases with the increase in the travel speed from 200 mm/min to 600 mm/min. This is because a faster travel speed reduces the frictional heat received in the repair area, reducing the grain growth and the dissolution of the second phase.

**Figure 14:**
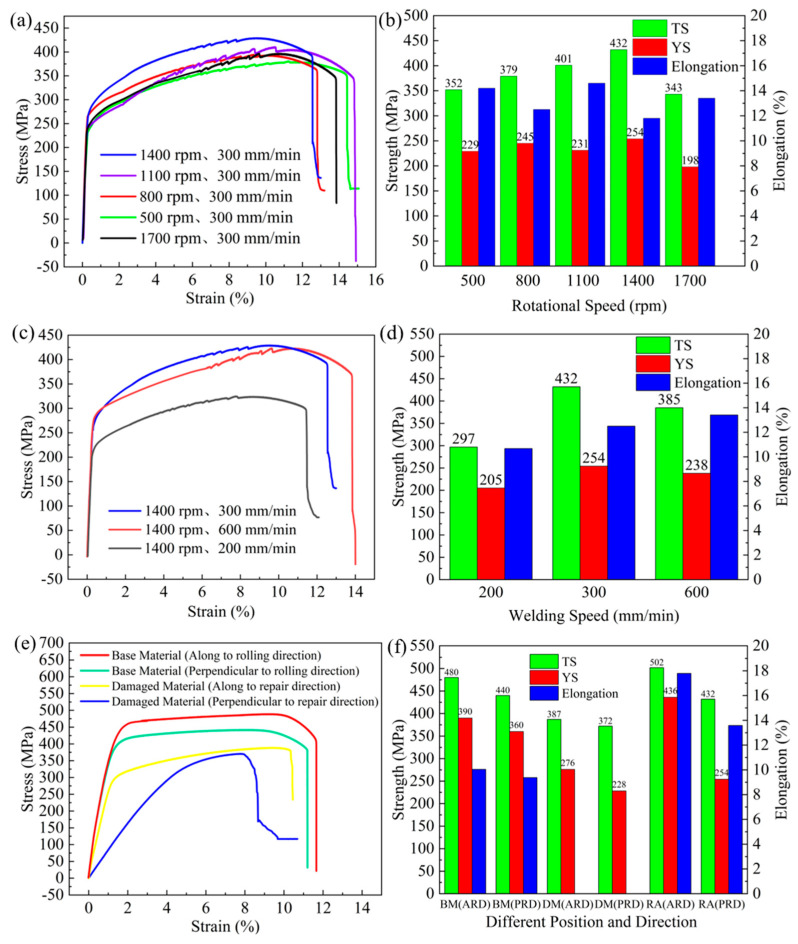
Stress–strain graphs with histograms of yield strength, tensile strength, and elongation of the specimens based on different parameters perpendicular to the repair direction: (**a**,**b**) gradual increase in rotational speed; (**c**,**d**) gradual increase in travel speed; (**e**,**f**) mechanical properties of the defects before repair and base material along different directions.

Figure 14 shows the stress–strain graphs with histograms of the yield strength, tensile strength, and elongation of the specimens according to different parameters perpendicular to the repair direction. The yield strength, tensile strength, and elongation are 254 MPa, 432 MPa, and 11.8%, respectively, at a rotational speed of 1400 rpm and a travel speed of 300 mm/min, which are about 111.4%, 116.1%, and 268.2% of those before the defects were repaired, respectively. When the preset defect exists, the yield strength, tensile strength, and elongation are only 228 MPa, 372 MPa, and 4.4%, respectively, which are seriously reduced compared with the base material. The experiments proved that the properties of the material are improved after the repair.

From Figure 14a, it can be seen that with an increase in the rotational speed, the tensile strength of the joint is gradually increased, while the elongation is gradually reduced, and an excessive rotational speed results in a reduction in the strength. Figure 14c shows that with an increase in the travel speed, the tensile strength of the material is increased, and the elongation is also increased, but an excessive rotational speed also causes a reduction in the properties of the repaired area. This is because, on the one hand, the excessive friction heat leads to an excessive increase in the plasticity of the material; under the top pressure and tangential speed of the tool, the flash causes the loss of material and the reduction in the NZ thickness. On the other hand, the excessive friction heat leads to the growth and coarsening of the recrystallized grains, resulting in the reduction in properties.

**Figure 15:**
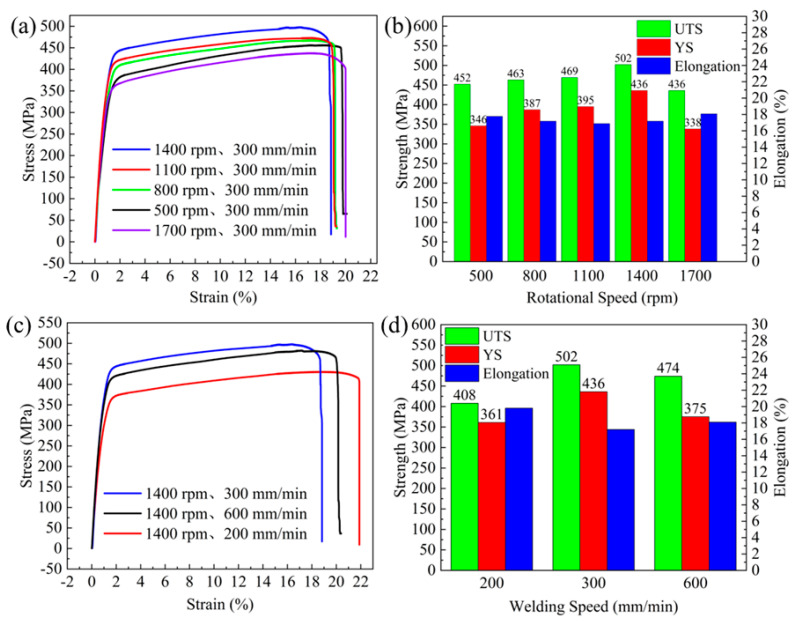
Stress–strain graphs with histograms of yield strength, tensile strength, and elongation for specimens according to different parameters parallel to the repair direction: (**a**,**b**) gradual increase in rotational speed; (**c**,**d**) gradual increase in travel speed.

Compared with those along the vertical repair direction, the properties along the repair direction are improved, as shown in Figure 15. When the repair parameters were 1400 rpm rotational speed and 300 mm/min travel speed, the yield strength, tensile strength, and elongation along the repair direction were 436 MPa, 502 MPa, and 17.2%, respectively, which were 157.9%, 129.7%, and 163.8% of the defects before repair, respectively. Both the rotational speed transformation and travel speed transformation showed a strong tendency of first increasing and then decreasing.

Analyzing the reason for the strengthening of the repair area, it is mainly that the NZ is subjected to plasticity and frictional heat; not only does the complete dynamic recrystallization occur but also some of the second phase presents an incoherent lattice with the base, which will be dissolved and form a new precipitation phase with the other phases after cooling, which improves the performance of the NZ. Analyzing the reasons for the fracture in the repair zone, on the one hand, it was found that dislocation accumulation occurs in the HAZ at the junction with the NZ, which generates stress concentration and is prone to crack; on the other hand, grain abnormality grows due to the influence of the friction heat only and the dissolution of the second phase occurs, which results in the decreased performance in this region, so the fracture zone is located at the junction of the NZ and the HAZ on the advancing side.

**Figure 16:**
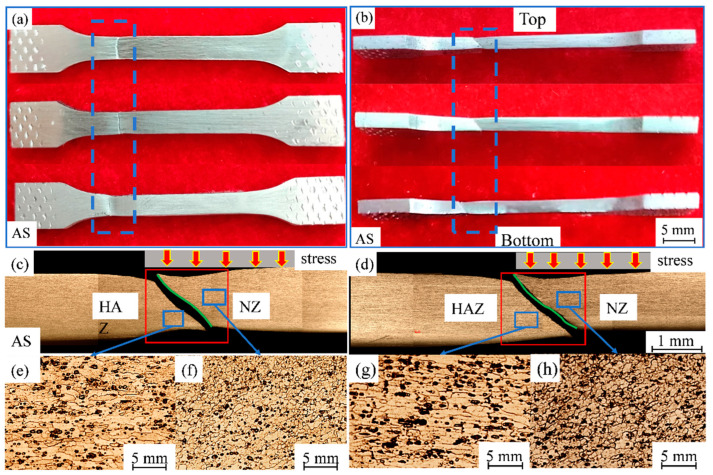
Macrograph and micrograph of fracture location of tensile samples for different parameters: (**a**,**b**) macrographs of the side and front view of the fracture; (**c**,**d**) micrographs of the fracture boundary under travel speeds of 300 rpm and 200 rpm, respectively; (**e**–**h**) microstructure of HAZ and NZ, respectively.

Figure 16 shows the macrograph and the microstructure of the fracture location of a typical tensile sample according to different parameters. It is observed that the fracture positions are all located at the junction of the HAZ and the NZ on the advancing side, and this phenomenon is consistent with the hardness measurements. In this study, the lateral macrographs clearly show that the samples all exhibit a shear fracture mode and the cracks fracture along the TMAZ-NZ boundary line, with both sides of the interface showing a completely different grain structure morphology.

**Figure 17:**
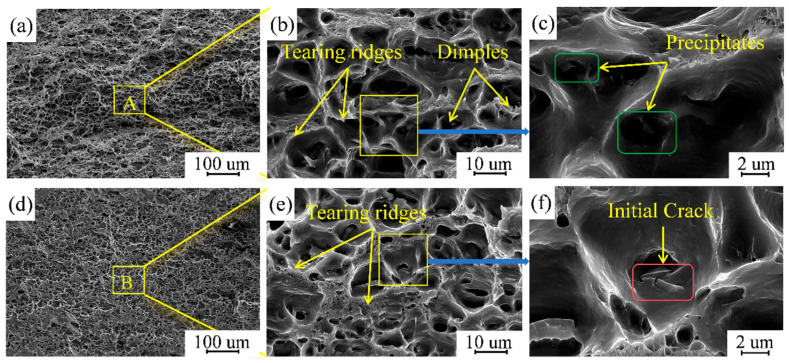
(**a**,**d**) SEM micrographs at the fracture of the specimens at travel speeds of 300 mm/min and 200 mm/min, respectively; (**b**,**e**) structure of tearing ridges and dimples at the fracture; (**c**) precipitated phase at the bottom of the dimples; (**f**) cracking source at the bottom of the dimples.

Figure 17 shows the SEM images of the fracture of the tensile samples according to different parameters. It can be observed that there are obvious dimple structures distributed in Figure 17a,d, and a certain amount of fine equiaxial dimples are contained inside the large dimples, which are small and deep, arising from ductile fracture [37]. Fine precipitates can be observed at the bottom of the dimple fossa, as shown in the green area of Figure 17c, which are attributed to the precipitated strengthening phases. Such a fracture morphology ensures good fracture strength and elongation in the repaired area, and, therefore, the fracture form is typical of ductile fracture [33].

## 4. Conclusions

In this study, a pinless stirring head is used for the surface friction repair of a 7A52 aluminum alloy thin sheet, and its structure evolution and property change rule are analyzed, revealing the location of fractures and elucidating the toughening mechanism within the nugget zone. The main conclusions are as follows:

(1) Utilizing surface friction repair technology on a 2 mm thick 7A52 aluminum alloy can obtain a defect-free repair area. The optimum process parameters are a rotational speed of 1400 rpm and a travel speed of 300 rpm, and the yield strength, tensile strength, and elongation along the repair direction are 436 MPa, 502 MPa, and 17.2%, which are about 157.9%, 129.7%, and 163.8% of those before the defects were repaired, respectively. Perpendicular to the repair direction, the yield strength, tensile strength, and elongation are 254 MPa, 432 MPa, and 11.8%, which are 111.4%, 116.1%, and 214.6% of those before the defects were repaired, respectively.
(2) The weak point of the strength perpendicular to the repair direction is located at the junction of the advancing side nugget zone and heat-affected zone. As the fine grains after dynamic recrystallization in the nugget zone and the coarse grains grown by heat in the heat-affected zone cause stress concentration at the junction, it is easy to observe fracture sources, and the material properties perpendicular to the repair direction are reduced compared with the base material.
(3) The nugget zone weave exhibits excellent isotropy and a large number of recrystallized weaves {001} <110>; the heat-affected zone shows excellent anisotropy, there is still a residual rolled weave S {123} <634>, and the content and strength of the weave vary with different degrees of thermal coupling.
(4) The second phase in the nugget zone is mainly the Al_12_(Fe, Mn)_3_Si phase and the semicoherent interface Al_6_Mn phase. The Mg element in the Mg_2_Si phase is dissolved, in which the Si element forms the α-Al_12_(Fe, Mn)_3_Si phase with the Al_6_(Fe, Mn) phase. Meanwhile, the presence of the Al_6_Mn phase leads to an increase in the mechanical properties of the repaired area, which is important for the improvement of the mechanical properties of the material parallel to the repair direction compared to the base material.

## Figures and Tables

**Table 1:** Chemical composition of 7A52 aluminum alloy.

Element	Si	Fe	Cu	Mn	Mg	Cr	Zn	Ti	Al
wt.%	0.25	0.3	0.1	0.25	2.0	0.15	4.2	0.05	Bal.

**Table 2:** Welding parameters for surface friction repair.

Experimental Parameters	Numerical Value
Rotational speed (rpm)	500, 800, 1100, 1400, 1700
Travel speed (mm/min)	200, 300, 600
Tool tilt angle (°)	2.5
Press amount (mm)	0.15

**Table 3:** Type and percentage of texture in different regions for different parameters.

Color Code	{hkl} <uvw>	NZ (%) (300 mm/min)	HAZ (%) (300 mm/min)	NZ (%) (200 mm/min)	HAZ (%) (200 mm/min)
	{100} <012>	29.4	19.8	37.5	23.9
	{011} <100>	23.0		18.0	
	{110} <112>	14.3		15.4	
	{001} <110>	14.2	14.7		15.6
	{112} <111>			20.6	
	{123} <634>		31.6		25.7
	{112} <110>		17.5		14.7

## Data Availability

Data will be made available on request.
